# Exploring the response of rice (*Oryza sativa*) leaf to gibberellins: a proteomic strategy

**DOI:** 10.1186/1939-8433-6-17

**Published:** 2013-07-01

**Authors:** Xiaoqin Wang, Feng Han, Mingfeng Yang, Pingfang Yang, Shihua Shen

**Affiliations:** Key Laboratory of Urban Agriculture (North) Ministry of Agriculture, Beijing University of Agriculture, Beijing, 102206 China; Institute of Botany, Chinese Academy of Sciences, Beijing, 100093 China; College of Life Sciences, Northwest A&F University, Yangling, 712100 China; Wuhan Botanical Garden, Chinese Academy of Sciences, Wuhan, 430074 China

**Keywords:** Gibberellins, Proteome, Rice leaf, Leaf expansion, cdc48

## Abstract

**Background:**

Gibberellins (GAs) are plant-specific hormones that play a central role in the regulation of growth and development with respect to environmental variability. Plants respond to GAs signal through various biochemical and physiological processes. To better understand the response for GA signal, we carried out a proteomic study in rice (*Oryza sativa* L. spp. *japonica*) leaf.

**Results:**

Through two-dimensional gel electrophoresis (2-DE) and mass spectroscopy analysis, we identified 61 proteins as GA-responsive. These proteins were annotated in various biological functions, such as signal transduction and cell growth/division, photosynthesis and energy metabolism, protein stability and defense. Among these, photosynthetic proteins decreased while many catabolic proteins increased. In addition, GA up-regulated a variety of cell growth/division, protein stability and defense proteins such as cell division cycle protein 48, molecular chaperones, and catalases.

**Conclusion:**

This is the first report that cell division cycle protein 48 may be responsible for leaf expansion after leaf sensing GA signal. The results presented here provide new insight into the mechanism of rice leaf in response to GA signal.

**Electronic supplementary material:**

The online version of this article (doi:10.1186/1939-8433-6-17) contains supplementary material, which is available to authorized users.

## Background

As a sessile eukaryote, plants have evolved a fine mechanism that makes them sense and respond to the exterior-environmental changes accurately. For a long time, it was presumed that there are some specific substances that not only associate with the plant’s environmental responses, but also control various aspects of plant growth and development (Gazzarrini and McCourt 2003). This idea was proved to be true when the first plant hormone was identified to be involved in the regulation of many plant processes (Davies 1995).

Among all the phytohormones, gibberellins (GAs) are a large family of tetracyclic diterpenoid that act nearly at all stages of the plant’s development, including germination, hypocotyl and stem elongation, leaf expansion, flowering and seed development (Davies 1995). Because of these reasons, it is very important to understand the GA responding mechanism of plant especially crops. In recent years, the identification of GID1 (a GA receptor) in rice and Arabidopsis brought us a much better understanding about the molecular mechanisms of GA signal transduction (Nakajima et al. 2006; Ueguchi-Tanaka et al. 2005). But the study on the changes of biochemical and physiological processes after sensing the GA signal is still very limited. Functional genomics strategy, such as transcriptomics and proteomics, might be very helpful for us to get a whole idea about what happen in the plant after sensing the signal of GA. Microarray analysis showed that the GA regulated genes have specific cis-elements at their upstream regions (Yazaki et al. 2003), and these genes fall into the functional groups of signal transduction, transcription, metabolism, cellular organization, and defense or anti-stress responses (Yang et al. 2004). Using proteomic techniques, it showed that calreticulin might be an important component in the GA signaling pathway that regulates rice seedling leaf-sheath elongation (Komatsu et al. 2006; Shen et al. 2003). They also showed that methylmalonate-semialdehyde dehydrogenase (MMSDH) may play a role in the development of root and elongation of leaf sheath in rice (Tanaka et al. 2005). Because of the limitation on both the techniques and genome sequence information, the proteins identified in both of the two studies are very limited. Moreover, they did not show us that biochemical and physiological changes of leaf expansion after sensing GA signal.

Leaf expansion is a complicated regulatory process which is involved in cell division and elongation (Avery 1993), and it has been suggested that the control of cell division and elongation are important factors in the regulation of growth and development (Vernoux et al. 2000). Previous studies have reported that cell division cycle proteins (CDC), cyclins and cyclin-dependent kinases (CDKs) are the important factors which play vital roles in regulating the cell cycle in eukaryotic organisms (Beemster et al. 2003; Inze and De Veylder 2006). The activity of CDKs can be regulated by other proteins through phosphorylation/dephosphorylation, direct binding or proteolysis (King et al. 1996; Morgan 1995). Different CDK-cyclin complexes phosphorylate a plethora of substrates at the key G1-to-S and G2-to-M transition points, triggering the onset of DNA replication and mitosis, respectively. For example, the phosphoprotein CDC34 catalyzes the covalent attachment of ubiquitin which regulates the G1/S transition of the cell cycle (Kaiser et al. 2000); the CDC2/CDC28 is a cyclin-dependent protein kinase that is required for both G1/S and G2/M transitions in yeast (Beach et al. 1982), whereas in higher organisms it is required for G2/M phase transition (Lessard et al. 1999). These studies showed that CDC and CDKs participate in the regulation of cellular cycle directly and thereby regulate cell size and cell number. Hence, we conjecture that CDC or CDKs may be responsible for rice leaf expansion after sensing the signal of GA.

In the present study, to uncover the changes of biochemical and physiological processes in rice leaf, we applied the proteomic analysis of rice seedlings in responding to GA_3_. Sixty-one proteins have been identified as being up- or down-regulated in response to GA_3_ treatment. These proteins are involved in signal transduction, cell growth/division, energy metabolism, protein stability, and defense responses, as well as others. These results might help to gain further information about the possible physiological and biochemical changes in rice leaf, hence contribute to the understanding of plant’s response to GA_3_ signals.

## Results

### GA content and morphological changes of rice leaf under application of GA_3_

**Figure 1:**
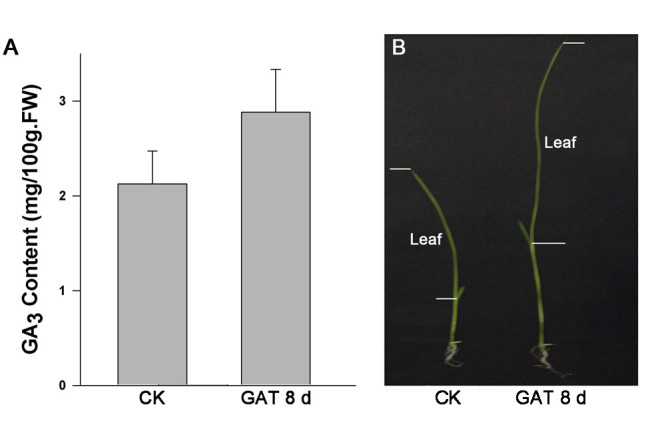
**GA content changes in rice leaf and leaf expansion changes in phenotype after application exogenous GA**_**3**_**. A,** The GA content of treatment is higher than that of control. Data is representative of three independent experiments and shown as mean + s.e. **B,** The leaf of treatment is significantly higher than that of control.

As mentioned above, GA can act as both the reproductive and vegetative stage of plant development including the expansion of plant leaf. After 8 days treatment with exogenous GA_3_, the GA content of the leaf showed significant increase (Figure 1A) and the rice seedlings grew at a higher rate than in normal conditions. The growth of the leaf increased dramatically in comparsion with the control (Figure 1B). These are consistent with the observations of rice sheath as previous study has shown (Shen et al. 2003).

### Chlorophyll content changes of rice leaf under application of GA_3_

**Figure 2:**
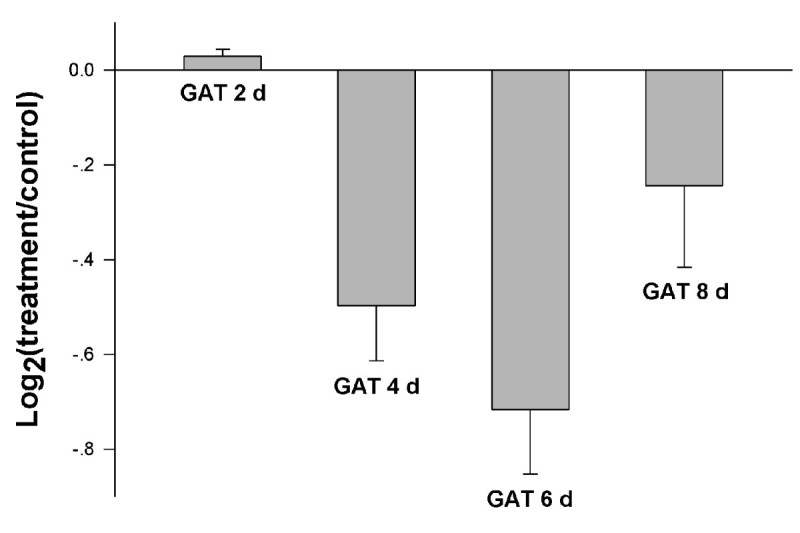
**Changes of the chlorophyll content of the rice treated by GA**_**3**_ **for 2, 4, 6 and 8 days.** Figure plots means ± SD from three replicate experiments.

Leaf chlorophyll concentration is an important parameter that is frequently measured as an indicator of chloroplast development, photosynthetic capacity, or general plant health. During the application of GA_3_, chlorophyll content increased slightly for GA_3_ treatment 2 days, and then declined for GA_3_ treatment 4, 6, 8 days (Figure 2).

### Proteome profile of rice leaf and its changes under application of GA_3_

**Figure 3:**
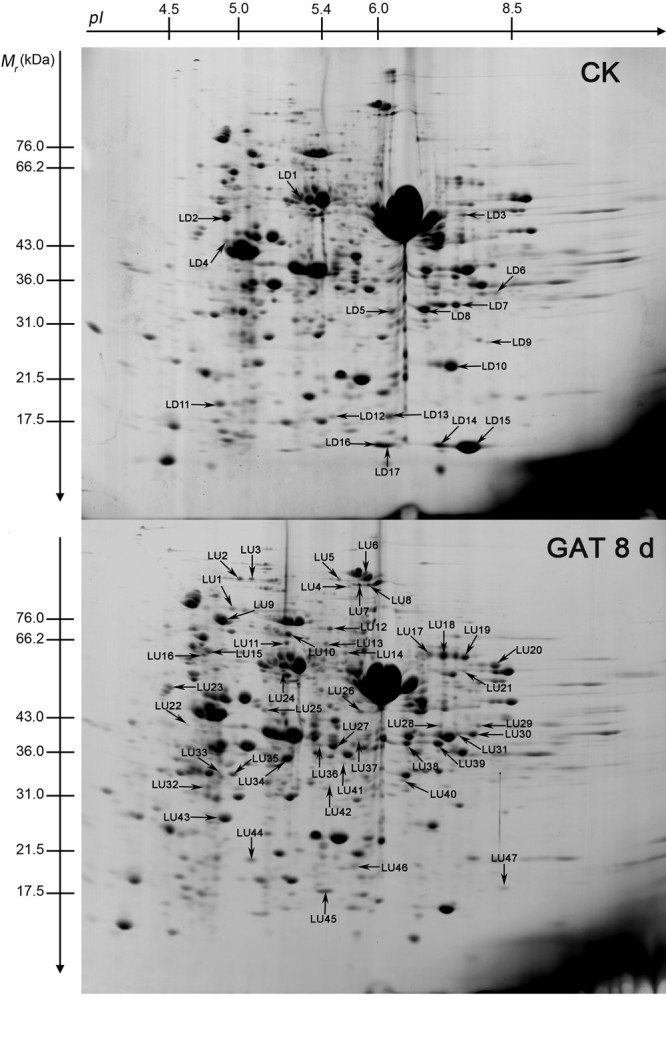
**Representative 2-DE gels of rice leaf proteins after application exogenous GA**_**3**_**.** Upper image: Control; lower image: **GA**_**3**_ treatment 8 days. GA-responsive proteins are indicated as follows: **LD,** down-regulated protein; **LU,** up-regulated protein. Gels are coommassie stained.

In order to uncover the correlation between the increased growth of rice seedlings and biochemical and physiological changes after application of exogenous GA_3_, total protein were extracted from the rice leaf and resolved by 2-DE. The experiments (from plants’ treatment to 2-DE) were carried out in three replicates for each sample. The leaf proteome was established over the *pI* range from 3.5 to 8 and molecular mass range from 10 to 100 kDa. More than 1000 protein spots could be resolved reproducibly on each gel. Representative gels from the control and treatment plants are shown in Figure 3.

**Figure 4:**
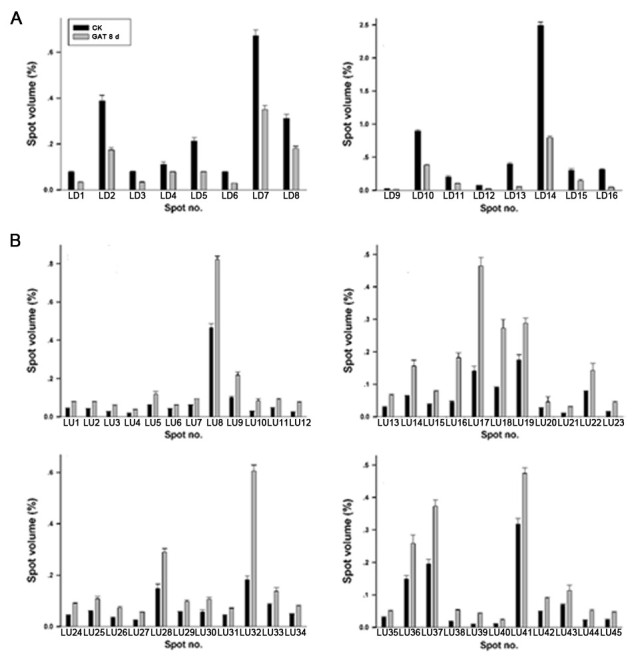
**Expression changes of the differentially displayed proteins. A,** Proteins down-regulated in response to GA_3_ as identified in Figure 2; **B,** Proteins up-regulated in response to GA_3_ as identified in Figure 2. The protein abundance is presented as the percentage of the total spot volume associated with each identified spot. Data is representative of three independent experiments and shown as mean + s.e.

Comparative analysis showed that there were 61 differentially displayed protein spots whose abundance altered at least 1.5-fold (Student’s *t* test, p < 0.05) relative to the control. Among these identified protein spots, sixteen were down-regulated (LD1 to LD16), and forty-five were up-regulated (LU1 to LU45) (Figure 3). Quantitative changes of these proteins are shown in Figure 4.

The response of exogenous GA_3_ is a complicated physiological process, in which many biochemical processes might be initiated or inhibited. So, it is reasonable to expect changes in the abundance of the proteins after GA_3_ treatment. Here, intensities of 61 out 1000 spots have been changed by more than 1.5-fold and these proteins are worthy of further study.

### Protein identification and functional categorization

To identify the differentially displayed proteins, the protein spots were excised from the 2D gels. After trypsin digestion, the peptides were extracted from the gel and analyzed by MALDI-TOF MS. Using the criteria described in Methods, we identified confidently 61 GA-responsive protein spots (Table 1). We observed some differences between the experimental and theoretical MWs and pIs of the proteins we identified. This may be due to post-translational modification of the proteins.

**Table 1:** **Identification of GA-responsive proteins in rice leaf**

Protein ID	Accession no.	Theor Mr (kD)/pI	Exp. (kD)/pI	Description	Matched/searched peptides	Score	Sequence coverage (%)	Metabolic group
LD1	AAA84588	54.0/5.30	57.6/5.29	AtpB gene product	14/46	139	51	Unknown
LD2	BAC78572	51.8/5.43	51.4/4.91	Ribulose-bisphosphate carboxylase activase large isoform precursor protein	13/28	142	37	Energy
LD3	BAD20105	30.3/11.81	52.5/7.65	Hypothetical protein	7/41	64	37	Unknown
LD4	EAZ34180	8.02/6.84	45.3/4.92	Hypothetical protein OsJ_017663	4/14	71	54	Unknown
LD5	CAG34174	53.3/6.23	31.3/6.26	Ribulose bisphosphate carboxylase large chain	11/77	66	20	Energy
LD6	BAD38069	22.3/9.97	34.8/8.24	Hypothetical protein	10/71	73	54	Unknown
LD7	CAG34174	53.3/6.23	32.3/7.46	Ribulose bisphosphate carboxylase large chain	9/35	79	21	Energy
LD8	BAD35346	13.7/10.25	31.8/6.89	Hypothetical protein	5/29	62	52	Unknown
LD9	ABA99483	120.3/8.37	26.8/8.07	Retrotransposon protein, putative, unclassified	10/28	65	13	Transposons
LD10	BAA31953	29.5/8.41	23.7/7.37	Carbonic anhydrase	6/34	64	30	Energy
LD11	O22386	18.6/5.36	19.3/4.86	50S ribosomal protein L12, chloroplast precursor (CL12)	5/26	66	40	Protein stability
LD12	ABA99483	120.3/8.37	18.0/5.53	Retrotransposon protein, putative, unclassified	10/28	65	13	Transposons
LD13	AAR19268	19.8/9.03	15.6/7.19	Ribulose-1,5-bisphosphate carboxylase/oxygenase small subunit	12/41	149	62	Energy
LD14	AAR19268	19.8/9.03	15.2/7.69	Ribulose-1,5-bisphosphate carboxylase/oxygenase small subunit	11/28	145	57	Energy
LD15	AAR19268	19.8/9.03	15.5/5.98	Ribulose-1,5-bisphosphate carboxylase/oxygenase small subunit	13/43	166	70	Energy
LD16	AAR19268	19.8/9.03	15.5/6.16	Ribulose-1,5-bisphosphate carboxylase/oxygenase small subunit	11/26	164	57	Energy
LU1	AAB63469	73.7/5.30	79.2/4.91	Endosperm lumenal binding protein	7/10	90	15	Unknown
LU2	AAP53974	90.5/5.09	92.3/5.02	Cell division cycle protein 48, putative, expressed	16/40	118	22	Cell growth/division
LU3	AAP53974	90.5/5.09	92.8/5.07	Cell division cycle protein 48, putative, expressed	20/47	158	27	Cell growth/division
LU4	ABB47613	92.1/6.28	89.6/5.70	Prolyl oligopeptidase family, putative	15/68	83	28	Unknown
LU5	BAD35509	112.4/6.35	95.1/5.89	Putative glycine dehydrogenase	12/22	124	20	Metabolism
LU6	BAC75578	15.5/10.82	89.6/5.82	Hypothetical protein	5/26	65	51	Unknown
LU7	NP_001052057	95.0/5.85	89.6/5.92	Os04g0118400	11/26	106	20	Unknown
LU8	AAX95352	71.5/5.10	74.1/4.89	DnaK-type molecular chaperone hsp70 - rice (fragment)	15/45	96	23	Protein stability
LU9	BAD82294	60.9/5.42	68.9/5.24	Putative phosphoglycerate mutase	14/43	105	30	Energy
LU10	AAR01748	42.2/6.10	65.8/5.24	Methylenetetrahydrofolate reductase, 3-partial	7/23	67	24	Metabolism
LU11	CAE05155	75.4/5.83	65.8/5.48	OSJNBa0039C07.11	12/21	129	22	Unknown
LU12	NP_001043066	62.9/5.68	62.3/5.44	Os01g0372700	15/39	126	26	Unknown
LU13	NP_001049057	60.6/7.25	62.6/5.66	Os03g0163300	10/54	72	25	Unknown
LU14	AAX85991	57.1/4.95	62.8/4.79	Protein disulfide isomerase	15/72	98	34	Protein stability
LU15	AAX85991	57.1/4.95	61.7/4.75	Protein disulfide isomerase	9/34	63	20	Protein stability
LU16	CSRZ	57.1/6.75	62.3/6.99	Catalase (EC 1.11.1.6) catA	7/25	69	23	Defence
LU17	CSRZ	57.1/6.75	61.7/7.25	Catalase (EC 1.11.1.6) catA	19/53	176	51	Defence
LU18	CSRZ	57.1/6.75	61.4/7.65	Catalase (EC 1.11.1.6) catA	12/38	92	27	Defence
LU19	BAC84709	60.6/7.19	58.2/8.20	Receptor protein kinase-related protein-like	6/22	73	15	Signal transduction
LU20	AAM12499	55.2/5.95	57.0/7.63	ATPase alpha subunit	10/42	120	30	Energy
LU21	BAD53795	46.7/5.81	42.8/4.64	Putative aminolevulinate dehydratase	11/70	74	35	Metabolism
LU22	ABF99934	37.3/4.64	52.1/4.51	Ankyrin repeat domain protein 2, putative, expressed	11/35	132	45	Metabolism
LU23	ABD57308	51.8/5.43	56.8/5.22	UDP-glucose pyrophosphorylase	11/59	77	35	Energy
LU24	NP_001047463	45.4/5.98	46.5/5.13	Os02g0621700	16/56	147	48	Unknown
LU25	AAO23563	46.0/5.90	45.8/5.82	Aspartate aminotransferase	21/58	213	59	Metabolism
LU26	EAZ41273	42.0/8.46	42.6/7.18	Hypothetical protein OsJ_024756	7/37	65	24	Unknown
LU27	BAB84334	39.5/7.16	42.6/7.89	UDP-glucuronic acid decarboxylase	11/57	90	44	Energy
LU28	AAA82047	36.6/6.61	40.7/7.79	Glyceraldehyde-3-phosphate dehydrogenase	7/15	100	33	Energy
LU29	AAA82047	36.6/6.61	40.9/7.49	Glyceraldehyde-3-phosphate dehydrogenase	7/21	80	27	Energy
LU30	AAX96859	51.6/9.06	30.7/4.78	Acetolactate synthase, small subunit, putative	9/24	62	16	Metabolism
LU31	BAD35207	34.5/5.61	33.2/4.86	Putative PrMC3	14/41	157	46	Unknown
LU32	BAB71741	32.9/5.51	35.8/5.24	Glyoxalase I	4/16	75	27	Energy
LU33	NP_001060741	27.2/5.21	32.8/4.97	Os07g0694700	9/20	135	58	Unknown
LU34	AAL61542	33.5/5.69	38.5/5.39	Isoflavone reductase-like protein	7/25	85	42	Metabolism
LU35	BAD08085	39.9/6.37	39.5/5.81	Putative ornithine carbamoyltransferase	8/35	75	34	Metabolism
LU36	NP_001053139	36.9/6.34	38.7/6.57	Os04g0486600	8/20	108	39	Unknown
LU37	NP_001053139	36.9/6.34	38.7/7.14	Os04g0486600	12/56	104	45	Unknown
LU38	NP_001054439	31.4/9.13	31.9/6.52	Os05g0110300	7/34	67	22	Unknown
LU39	BAD25718	28.8/5.46	35.1/5.65	Putative porphobilinogen deaminase	7/48	64	35	Metabolism
LU40	ABA93708	85.5/8.06	31.1/5.48	NB-ARC domain, putative	11/31	64	16	Unknown
LU41	BAD36628	23.2/5.72	25.9/4.91	Putative chaperonin 21 precursor	14/42	181	74	Protein stability
LU42	BAB70686	79.6/5.37	20.9/5.07	Ryptochrome 1a	6/26	65	10	Unknown
LU43	BAD32104	24.8/11.92	17.8/5.44	Splicing coactivator subunit-like protein	6/28	65	28	Metabolism
LU44	NP_001042680	21.0/7.77	20.2/5.77	Os01g0266600	6/31	74	54	Unknown
LU45	EAZ35500.1	21.6/8.38	18.1/8.36	Hypothetical protein OsJ_018983	5/15	70	24	Unknown

**Figure 5:**
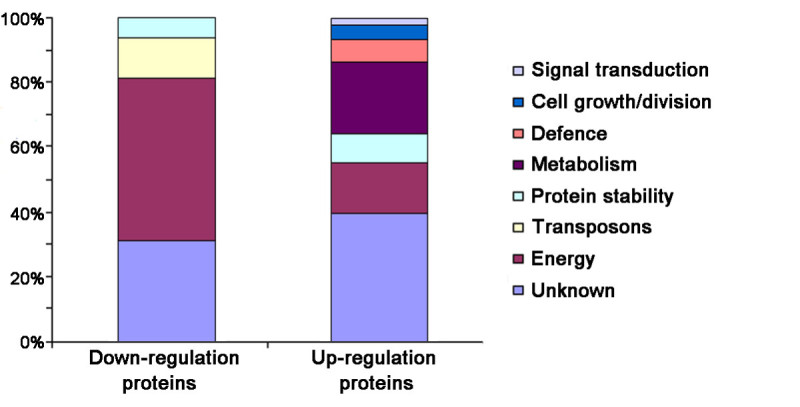
**Functional categorization of the GA-responsive proteins.** Seven groups were represented by up-regulated proteins and only four groups by down-regulated proteins.

The identified proteins could be sorted into 8 categories (Table 1, Figure 5) according to their function as described by the EU *Arabidopsis* Genome Project (Bevan et al. 1998). These functional classes included energy, metabolism, defense, protein stability, cell growth/division, signal transduction, and transposons (Table 1, Figure 5). Among them, proteins of the energy and metabolism groups accounted for more than 65.7% of the total number identified.

### Proteins down-regulated by GA_3_ treatment

Sixteen proteins were down-regulated following application of exogenous GA_3_. Seven of them were identified as a subunit of ribulose bisphosphate carboxylase/oxygenase (spots LD2, LD5, LD7, LD13, LD14, LD15 and LD16). They catalyze the first step in net photosynthetic CO_2_ assimilation and photorespiration carbon oxidation. Carbonic anhydrase (spot LD10), which plays a role in converting bicarbonate ions back to carbon dioxide for photosynthesis, is also identified. In addition, proteins related to transposons (spots LD9 and LD12) were also down-regulated. Our results suggest that the photosynthesis might be inhibited by application of exogenous GA_3_. This may lead to reduced energy of the cells.

### Proteins up-regulated by GA_3_ treatment

Forty-five proteins were up-regulated in response to application of exogenous GA_3_. Among them, more than 53.3% of the proteins belonged to four functional groups: energy, metabolism, protein stability and defense.

The proteins involved with energy and metabolism proteins were mainly linked to glycolysis, citric acid cycle, pentose phosphste pathway and polysaccharide synthesis, including glyceraldehyde-3-phosphate dehydrogenase (spots LU28 and LU29), phosphoglycerate mutase (spot LU9), UDP-glucuronic acid decarboxylase (spot LU27), UDP-glucose pyrophosphorylase (spot LU23) and ATPase alpha subunit (spot LU20). This suggests that energy metabolism is activated by application of exogenous GA_3_.

The proteins involved in defense and protein stability were the third most abundant category of up-regulated proteins. There were DnaK-type molecular chaperone hsp70 (spot LU8), disulfide isomerase (spots LU14, LU15), chaperonin 21 (spot LU41) and catalase (spots LU16, LU17, LU18). An important role of molecular chaperones (spots LU8 and LU41) is stabilizing protein conformation by preventing the aggregation of denatured or incompletely folded proteins and by promoting the re-naturation of aggregated proteins (Boston et al. 1996). Protein disulfide isomerase (spots LU14, LU15), an enzyme located in the endoplasmic reticulum, catalyzes the folding of many disulfide-bonded proteins (Freedman, 1984). Catalase (spots LU16, LU17, LU18) is a key antioxidant enzyme in the bodie’s defense against oxidative stress. It converts the reactive oxygen species hydrogen peroxide to water and oxygen and thereby mitigates the toxic effects of hydrogen peroxide. Their up-regulation suggests that defense is also enhanced as a response to application of exogenous GA_3_ in this plant.

Proteins associated with signal transduction (receptor protein kinase-related protein; spot LU19; Figure 2) and cell growth/division (cell division cycle protein 48, CDC48; spots LU2 and LU3) were also up-regulated. CDC48 is a highly abundant type II AAA-ATPase associated with two copies of the highly conserved ATPase domain, with each containing the consensus Walker A and B motifs which are responsible for ATP binding and hydrolysis, respectively (Buchberger 2010). Upon ATP binding and hydrolysis, CDC 48 undergo conformational changes, which could generate a pulling force to disassemble a protein complex such as mitotic spindle (Cheeseman and Desai 2004).

### Quantitative real-time PCR analysis of GA-responsive genes in *P. patens*

**Figure 6:**
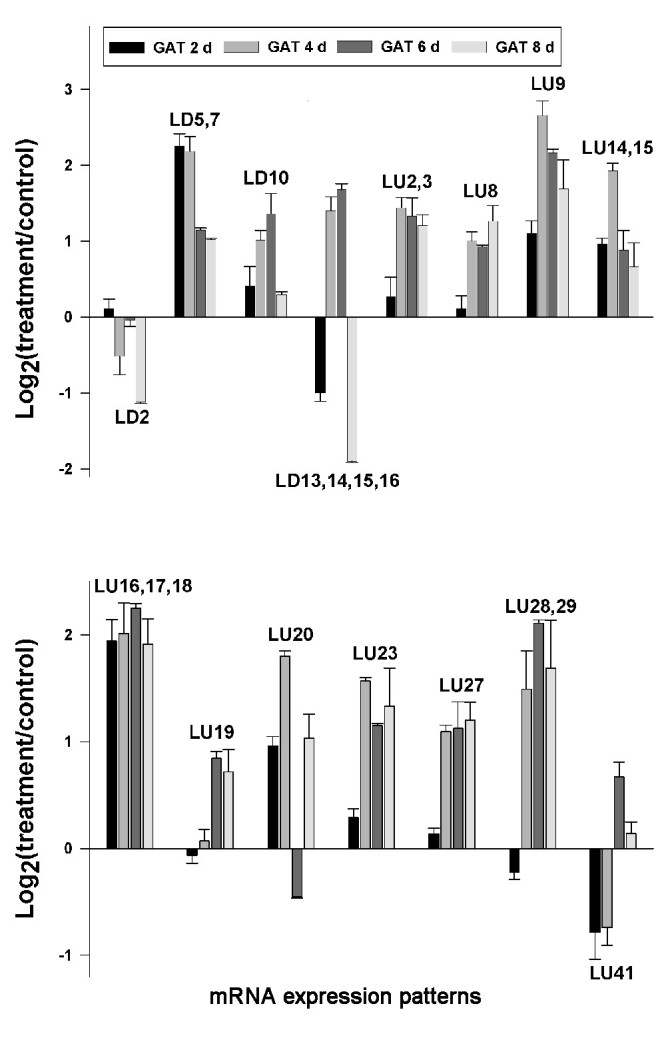
**mRNA expression patterns.** The log_2_ values were plotted as the relative expression of the rice treated by GA_3_ for 2, 4, 6 and 8 days.

To correlate protein level with the corresponding mRNA level of the GA-responsive genes, we performed quantitative real-time PCR. We analyzed the expression of 15 genes which were identified as GA-responsive genes. Except for a few genes, mRNA levels of most genes changed in parallel with protein levels (Figure 6). The parallel and independent relations that exist between mRNA and protein levels among GA-responsive genes imply the existence of a fairly complex regulatory network.

## Discussion

Because of its positive effects on plants, GAs have been regarded as a major plant hormone for controlling growth and development. It is well established that GAs are responsible for triggering stem or internodal elongation. In rice seedlings, the basal part of the leaf sheath contains root primodia and meristem cells, which undergo active cell division and development (Yang et al. 2005). Proteomic studies have focused on the mechanism of rice leaf sheath elongation following GA_3_ treatment. They have been shown that calreticulin is an important component in regulating rice seedling leaf sheath elongation. Further, the GA_3_ can induce the synthesis of many proteins in rice leaf sheath, including chaperones, metabolic enzymes, and detoxification enzymes (Komatsu et al. 2006; Shen et al. 2003; Tanaka et al. 2004). However, the study on the leaf expansion after sensing the GA signal is still very limited. In this work, the GA_3_-induced changes in the proteome of rice leaf were analyzed after treatment with GAs for 8 days. This study can help to reveal the mechanism of leaf expansion in response to GAs.

### Signal transduction and cell growth/division

Studies have shown that plant cells sense signal at the plasma membrane and then initiate signal transduction to regulate the expression of a series of relevant genes. Receptor protein kinases are plasma membrane-bound and play an important role in the perception and transmittance of external signals such as ABA, dehydration, high salt and cold treatments (Hong et al. 1997). In our study, receptor protein kinase-related protein-like (spot LU19) was up-regulated, implying that it was responsible for GA signal transduction and in turn aroused a series of physiological and biochemical changes. Plant morphological development requires the coordination of cell division, expansion, and differentiation (Beemster et al. 2003; Fleming 2006; Meijer and Murray 2001). It is known that GA can stimulate leaf expansion by an increase in cell length and cell number (Yang et al. 1996), with the promotion in cell extension being largely conferred by an enhanced wall extensibility (Cosgrove and Sovonick-Dunford 1989). Previous studies have been reported that cell division cycle protein 48 (CDC48; spots LU2 and LU3) may be directly involved in cell expansion, cell division and cell proliferation (Park et al. 2008; Rancour et al. 2004). Up-regulation of CDC48 by application exogenous GA_3_ indicates that CDC48 may be responsible for rice leaf expansion.

### Photosynthesis inhibition

A major effect of GAs on plant seedlings has been reported as producing increased rates of growth in size and weight. Such growth might be due to an increased rate of photosynthesis. However, the study of photosynthesis in several species indicated that GA did not enhance the rate of CO_2_ fixation per unit of leaf tissue (Haber and Tolbert 1957), and that photosynthesis was unaffected during leaf expansion (Haag-Kerwer et al. 1999). In our study, 7 out of 16 down-regulated spots in the group belonging to photosynthetic enzymes were identified (spots LD2, LD5, LD7, LD10, LD13, LD14, LD15 and LD16). During the application of GA_3_, chlorophyll content decreased for GA_3_ treatment 4, 6, 8 days (Figure 2). These suggest that photosynthesis may be inhibited following the application of exogenous GA_3_. This is consistent to several studies on plant photosynthesis in response to GA (Dijkstra et al. 1990; Thetford et al. 1995).

### Catabolic metabolism enhanced

In contrast to the apparent decrease of photosynthesis, the catabolic metabolism seems to be enhanced after application of exogenous GA_3_, or at least poised for enhancement, as seen from the increase in several energy related proteins (spots LU28, 29, LU9, LU27, LU23, and LU20). These enzymes are involved in energy metabolism pathway such as glycolysis, citric acid cycle, and pentose phosphate pathway and polysaccharide synthesis. Glycolysis and citric acid cycle are the main pathway for organisms to produce energy; the pentose phosphate pathway meets the need of all organisms for a source of NADPH to use in reductive biosynthesis; polysaccharide can store energy for organisms growth and development. These results may reflect that exogenous GA_3_ can accelerate energy in more than one way: it not only enhance energy directly by up-regulating ATPase alpha subunit (spot LU20), but also affects catabolic metabolism pathway. The same results have been reported in previous studies (Fu et al. 2005; Tanaka et al. 2004; Yang et al. 2004).

### Protein stability assurance

Protein stability related proteins were also identified in response to exogneous GA_3_. Molecular chaperones (spots LU8, LU41) are such proteins that can assist in *de novo* protein folding, stabilize proteins under stress conditions and maintain polypeptide chain components in a loosely folded state for translocation across organellar membranes (Hartl, 1996; Wang et al., 2004). Our data demonstrated that the expression of molecular chaperone proteins was enhanced in response to exogenous GA_3_, suggesting that GA_3_ increased a potential capacity of protein stability and cellular stress response. A pair of protein stability related proteins up-regulated by application exogenous GA_3_ are protein disulfide isomerase (PDI; spots LU14 and LU15). It also can assist the protein refolding to its active state by suppressing aggregation, which is closely similar to the action of chaperones (Cai et al. 1994). Together, it seems that plants have evolved protective mechanisms to adjust to cell expansion.

### Accumulation of defense proteins

Along with above-mentioned components of energy metabolism and protein stability, exogenous GA_3_ also up-regulated several proteins associated with defense against stress. Catalase (spots LU16, LU17 and LU18) is such an antioxidant enzyme from many species known to rapidly convert hydrogen peroxide into oxygen and water. In this study, the possible explanations for up-regulation of catalase may be as follows. First, the reduction of photosynthesis and enhancement of respiration will increase the formation of reactive oxygen species and hence increase the accumulation and activity of catalase that detoxifies hydrogen peroxide (Pellinen et al. 2002). Second, leaf expansion is corrected with cell wall loosening which is generally assumed to involve the scission of plant cell wall polysaccharides (Fry 1998). Along with the scission of cell wall polysaccharides, the hydroxyl radicals (•OH) which is produced by the reaction of hydrogen peroxide (H_2_O_2_) with oxygen (O_2_) have been shown to increase (Fry 1998). Our study showed that up-regulation of the catalase may be responsible for scavenging H_2_O_2_ generated by cell wall-loosening during leaf expansion. Therefore, it is not surprising that the expression of enzymes related to anti-oxidation was enhanced significantly in response to application exogenous GA_3_. Previous study has shown the same result in rice leaf sheath (Komatsu et al. 2006).

## Conclusions

**Figure 7:**
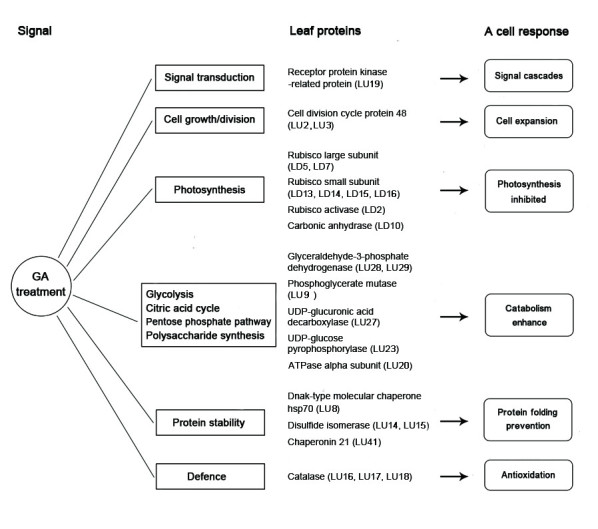
**Cell responses corresponding to identified proteins under the treatment by GA**_**3**_ **.**

This study presents a comprehensive analysis of rice leaf proteome in response to exogenous GA_3_ (Figure 7). Upon the application of exogenous GA_3_, rice leaf cells can sense GA_3_ and transmit a signal, which in turn, activates cell growth/division, energy metabolism, protein stability and defensive genes expression. These results should serve as useful starting points to develop a complete understanding of how the plant cell responds to GA_3_ signal.

## Methods

### Plant material and GA_3_ treatments

Rice (*Oryza sativa* L. spp. Japonica var. Nipponbare) seeds were allowed to germinate in distilled water for 48 h at 26°C. The germinated seeds were sown in beakers containing complete kimura B nutrient solution. For GA_3_ treatments, 5 μM GA_3_ was added into the nutrient solution. Seedlings were cultured under white light (150 μmol photons/m^2^s; 14 h light/10 h dark) at 26°C in a growth chamber. After 8 days, the second leaves were cut down for protein extraction. For quantitative real-time PCR and chlorophyll measure, 5 μM GA_3_ was added to the nutrient solution when seedlings growth for 0, 2, 4, 6 days. After 8 days, the second leaves were cut down for RNA and chlorophyll extraction.

### GA_3_ extraction and measure

The GA_3_ was extracted from rice leaf according to Kelen et al., (2004) with some modification. In brief, a half gram of leaf was ground into fine powder in liquid nitrogen. The powder was mixed with 30 ml ice-cold methanol for 1 h and then put into fridge (4°C) overnight. The mixture was strained through filter paper. The GA_3_ was reextracted from the residue as mentioned above. The liquid phase (methanol containing hormones of GA_3_) was merged and evaporated to 10 ml under vacuum. All the samples were filtered through 0.45 μm membrane (Millipore) using a filtration syringe system for analysis.

The content of GA_3_ was determined using high-pressure liquid chromatography (HPLC). Liquid chromatography was performed using Waters LC (Model 244) equipped with reversed phase column Diamosil C18 (250 mm × 4.0 mm). The mobile phase was methanol-acetonitrile-water (20/15/65; v/v), and pH was adjusted to 3.0 using 0.1 M H_3_PO_4_. The injection volume was 10 μl, the column temperature was set to 30°C, and the flow rate was 0.8 ml/min. The signal of the compound was monitored at 254 nm for GA_3_. The standard solution of GA_3_ was made in the mobile phase and chromatographed separately to identify retention time of GA_3_. The experiment was carried out in three repeats and the results were exported to SPSS Version 13.0 for statistical analysis using t- test (P < 0.05).

### Determination of chlorophyll content

To determine chlorophyll content, freshly harvested samples were extracted in a one to one mixture of acetone and ethanol, as described by Chen (1984), and the absorbance of the extract samples were measured at 652 nm with a spectrophotometer (GE Healthcare BIO-Science). Chlorophyll content was calculated based on Arnon (1949).

### Protein extraction and 2-DE

Proteins were extracted under a denaturing condition according to the procedure (phenol extraction procedure) described by Wang et al. (2009a) with minor modification. One g of leaf was ground into fine powder in liquid nitrogen and homogenized on ice with 2 ml ice-cold extraction buffer (250 mM sucrose, 20 mM Tris–HCl pH 7.5, 10 mM EDTA, 1 mM PMSF, and 1 mM DTT). Then an equal volume of ice-cold Tris–HCl pH 7.5-saturated phenol was added, and the mixture was re-homogenized on ice. After centrifugation (20 min, 15,000 g, 4°C), the phenol phase was collected. Proteins were precipitated from the final phenol phase with three volumes of 100 mM ammonium acetate in methanol overnight at −20°C. The pellets were rinsed three times with ice-cold acetone containing 13 mM DTT and then lyophilized. The resulting pellets were dissolved in a sample buffer (7 M urea, 2 M thiourea, 4% (w/v) CHAPS, 2% (v/v) Ampholine pH 3.5-10, 1% (w/v) DTT) at room temperature. Two-dimensional electrophoresis was carried out according to Yang et al. (2007). The isoelectrofocusing (IEF) gels were 15 cm long with a diameter of 3 mm. The gel solution contains 8 M urea, 3.6% (w/v) acrylamide, 2% NP-40 and 5% (v/v) Ampholines (1 part pH 3.5-10, 1 part pH 5–8). For each gel, 50 μl sample was loaded. IEF was performed at 200 V, 400 V and 800V for 30 min, 15 h and 1 h respectively. After the IEF run, IEF gels were equilibrated in equilibration buffer (62.5 mM Tris–HCl pH 6.8, 2.5% SDS, 10% (v/v) glycerol and 5% (v/v) 2-mercaptoethanol for 15 min twice. For the second dimension, the proteins were separated on 15% SDS polyacrylamide gels. Protein spots were visualized with Coomassie Brilliant Blue (CBB) R-250.

### Image and data analysis

The 2-DE gels were scanned at a 600 dots per inch (dpi) resolution with UMAX Power Look 2100XL scanner (Maxium Tech, inc., Taiwan, China). The transparency mode was used to obtain a grayscale image. The image analysis was performed with ImageMaster™ 2D Platinum version 5.0 (GE Healthcare BIO-Science). The optimized parameters were as follows: saliency 2.0, partial threshold 4 and minimum area 50. Spots were quantified on the basis of their relative volume (%V), which was determined by the ratio of the volume of a single spot to the whole set of spots. Only those with reproducible changes (quantitative changes more than 1.5-fold in abundance and t- test P < 0.05) among three replicates were used for further analysis.

### In-gel digestion and MALDI-TOF MS analysis

Protein spots were manually excised from the gel, and in-gel digestion by trypsin was performed according to Wang et al. (2009a) with some modifications. Gel slices were washed with 25% (v/v) ethanol and 7% (v/v) acetic acid for 12 h overnight at room temperature, and destained with 50 mM NH_4_HCO_3_ in 50% (v/v) methanol for 1 h at 40°C. Proteins were reduced with 10 mM DTT in 100 mM NH_4_HCO_3_ for 1 h at 60°C, and alkylated with 40 mM iodoacetamide in 100 mM NH_4_HCO_3_ for 30 min at room temperature in the dark. The gel pieces were minced and lyophilized, then digested with 10 ng sequencing-grade modified trypsin in 25 mM NH_4_HCO_3_ solution overnight at 37°C. After digestion, the peptides were collected, and the pellets were washed with 0.1% TFA in 50% v/v acetonitrile three times to collect the remaining peptides. The solution containing eluted peptides was desalted by ZipTipC 18P™.

Tryptic peptide masses were measured with an AXIMA-CFR plus MALDI-TOF mass spectrometry (Shimadzu Biotech, Kyoto, Japan). The acquired peptide-mass fingerprints (PMFs) were analyzed by searching NCBI database with the Mascot software available at (http://www.matrixscience.com). The searching parameters were set according to Yang et al. (2007). *O. sativa* was chosen for the taxonomic category and 0.2 Da was used as the mass error tolerance. To determine the confidence of the identification results, the following criteria were used: a minimum MOWSE score was 66, and sequence coverage of the protein should not be less than 12% by the matching peptides. Only the best matches with high confidence levels were selected.

### Real-time quantitative RT-PCR

Real-time quantitative RT-PCR was carried out as previously described (Wang et al. 2009b). Total RNA was extracted using the RNA PCR kit (Takara Bio, Otsu, Japan), and treated with DNAse-I (Ambion, Austin, TX), according to the manufacturer’s instructions. First strand cDNA was synthesized using the SMART PCR cDNA Synthesis Kit (BD Biosciences) according to the manufacturer's instructions. The *O. sativa* 18S rDNA gene was used as a standard to normalize the content of cDNA. PCR was performed using gene-specific primers (Additional file 1: Table S1) on a Rotor Gene 3000 Real-Time Thermal Cycler (Corbet Research, Sydney, Australia). SYBR Premix Ex Taq (Perfect Real Time) kit and RT-PCR reagents (Takara Bio) were used for quantification of differentially expressed gene sequences.

## Electronic supplementary material

Additional file 1: Table S1: Primer pairs used in Quantitative Real-Time PCR. (DOC 34 KB)

Below are the links to the authors’ original submitted files for images.

Authors’ original file for figure 1

Authors’ original file for figure 2

Authors’ original file for figure 3

Authors’ original file for figure 4

Authors’ original file for figure 5

Authors’ original file for figure 6

Authors’ original file for figure 7

## Abbreviations

CBB: Coomassie Brilliant Blue
CDC: Cell division cycle proteins
CDKs: Cyclins and cyclin-dependent kinases
2-DE: Two-dimensional gel electrophoresis
GAs: Gibberellins
GAT: GA_3_ treatment
HPLC: High-pressure liquid chromatography
IEF: Isoelectrofocusing
MMSDH: Methylmalonate-semialdehyde dehydrogenase
PMFs: Peptide-mass fingerprints.
