# Supplementary material for: Exploring the response of rice (Oryza sativa) leaf to gibberellins: a proteomic strategy
Source: Rice (N Y). 2013 Jul 1;6:17. doi: 10.1186/1939-8433-6-17 (PMC4883738; doi:10.1186/1939-8433-6-17)
Supplement: Supplementary file 1 — Additional file 1: Table S1: Primer pairs used in Quantitative Real-Time PCR. (DOC 34 KB) [file 12284_2013_54_MOESM1_ESM.doc]

**Supplemental Table1. Primer** pairs used in Quantitative Real-Time PCR

| **Spot No.** | **Forward Primer** | **Reverse Primer** |
| --- | --- | --- |
| LD2  LD5,7  LD10  LD13,14,  15,16  LU2,3  LU8  LU9  LU14,15  LU16,17,18  LU19  LU20  LU23  LU27  LU28,29  LU41  18S^(1)^ | 5"-CTGCTCTTGGTGACGCTAACTC  5"-GTGGAGGAACTTTAGGACATC  5"-ACTCATCGTGGTGATTGG  5"-AATGGCGGAAGGATCAAGTG    5"- TGTGGACTTGAACGCTCTTG  5"- CAGCGGACAAGAAGAAGA  5"- TCCGAGCCATTACCTTGTCT  5"- GGTGCTGCTGAGGAGTTC  5"- CGACGATACACAAGCAGAA  5"- AAGAAGAGGTGGTGGTGAA  5"- AACAGGAAGAATTGCTCAGATACC  5"- GGTCTCTGGTGATGTCTG  5"- GTCAGAGGAAGCCAGACATTACAA  5"- GCTGGAATTGCTCTTAACGATAAC  5"- CCACTAAGGAGAAGCCATCAATCG  5“-CTACGTCCCTGCCCTTTGTACA | 5"-TACCTGCTTGCTGTGCTCCTT  5"-CATTACCTTCACGAGCAAGA  5"-GAAGTGGAAGGAGTCTGG  5"-GGTGAGTGGTGGCAGGTA  5"- TTCTCCTTACTCCGCCTCTC  5"- CGAACTCATCAGCCTCAG  5"- GCCAAACTTCACTGTCTCACT  5"- CCTGGATGATGATGAGTGGTA  5"- TGAACCTCTCCTGTCTATCC  5"- AATCGGCGTACTGGAACT  5"- GCAGGAGATTCAATTAAGCGAGAT  5"- CGTTGATGTCCTTGTTCTC  5"- CACCAAGCCATCACGCAGAA  5"- CATGTGGCGGATCAGGTC  5"- GCTGCCTCTTGCCTTCATCA  5"-ACACTTCACCGGACCATTCAA |

1. 18S (Accession NO. AK059783)
