# Supplementary figures and images for: Exploring the response of rice (Oryza sativa) leaf to gibberellins: a proteomic strategy
Source: Rice (N Y). 2013 Jul 1;6:17. doi: 10.1186/1939-8433-6-17 (PMC4883738; doi:10.1186/1939-8433-6-17)

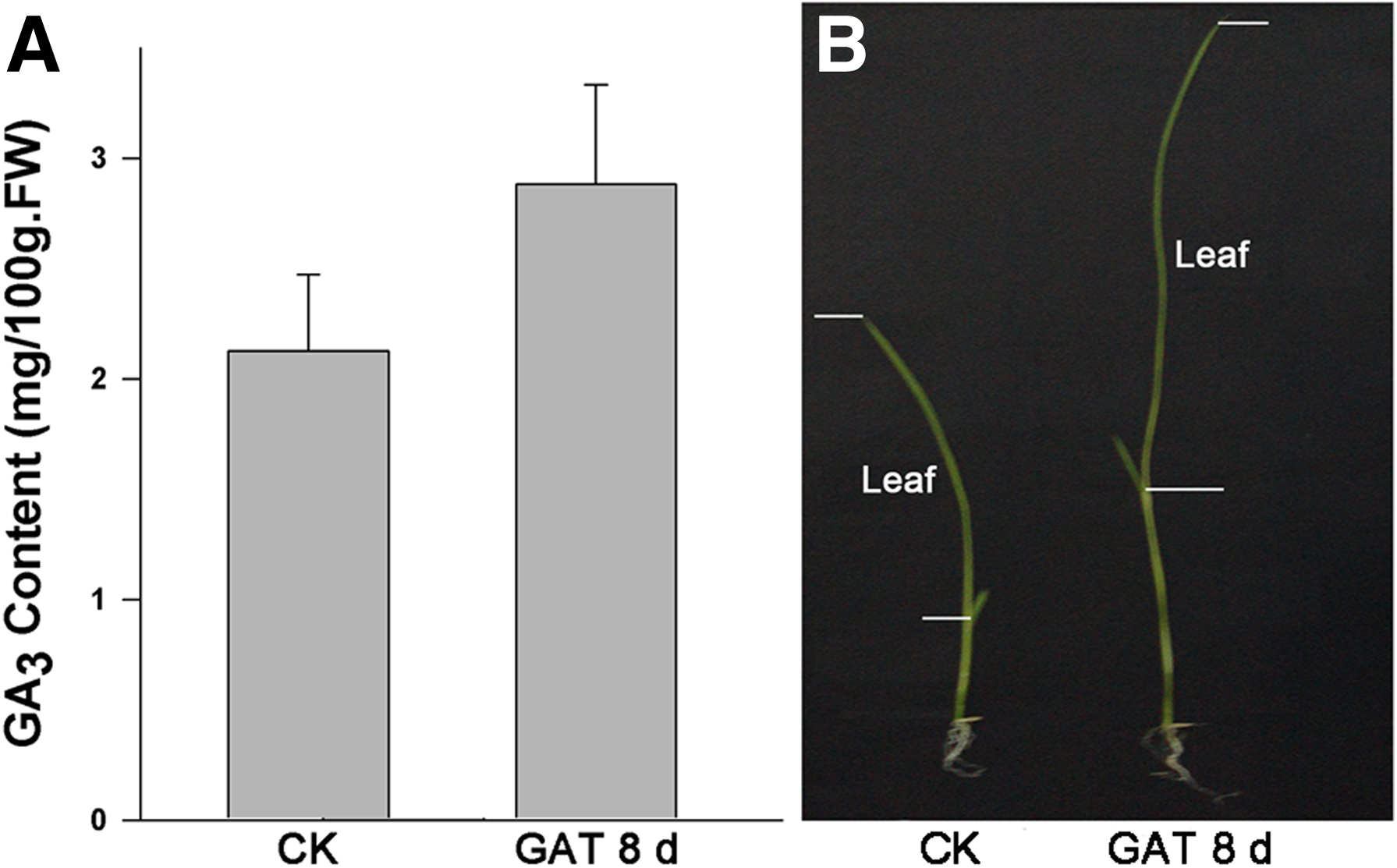

Supplement: Supplementary file 2 — Authors’ original file for figure 1 [file 12284_2013_54_MOESM2_ESM.tif]

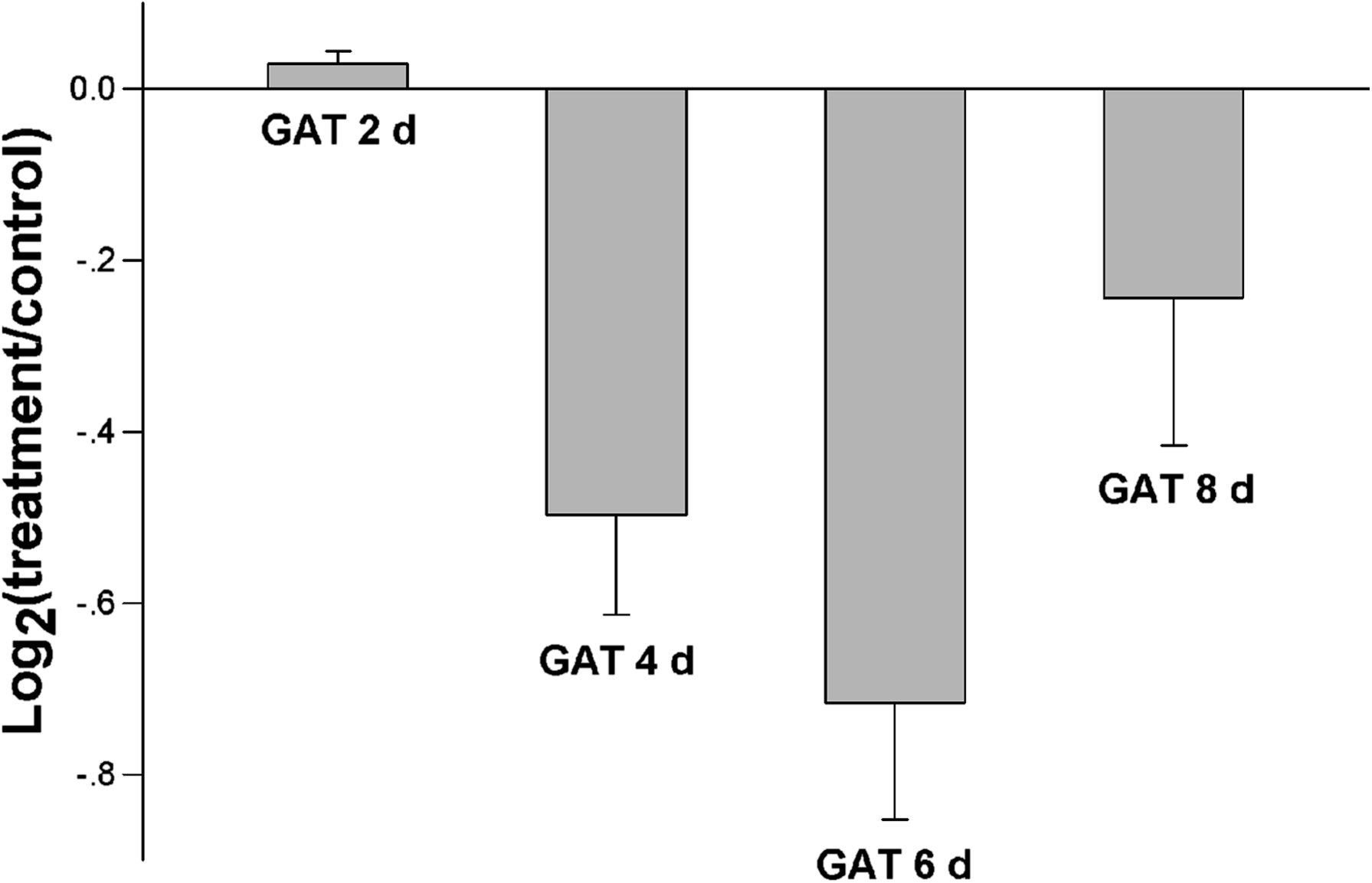

Supplement: Supplementary file 3 — Authors’ original file for figure 2 [file 12284_2013_54_MOESM3_ESM.tif]

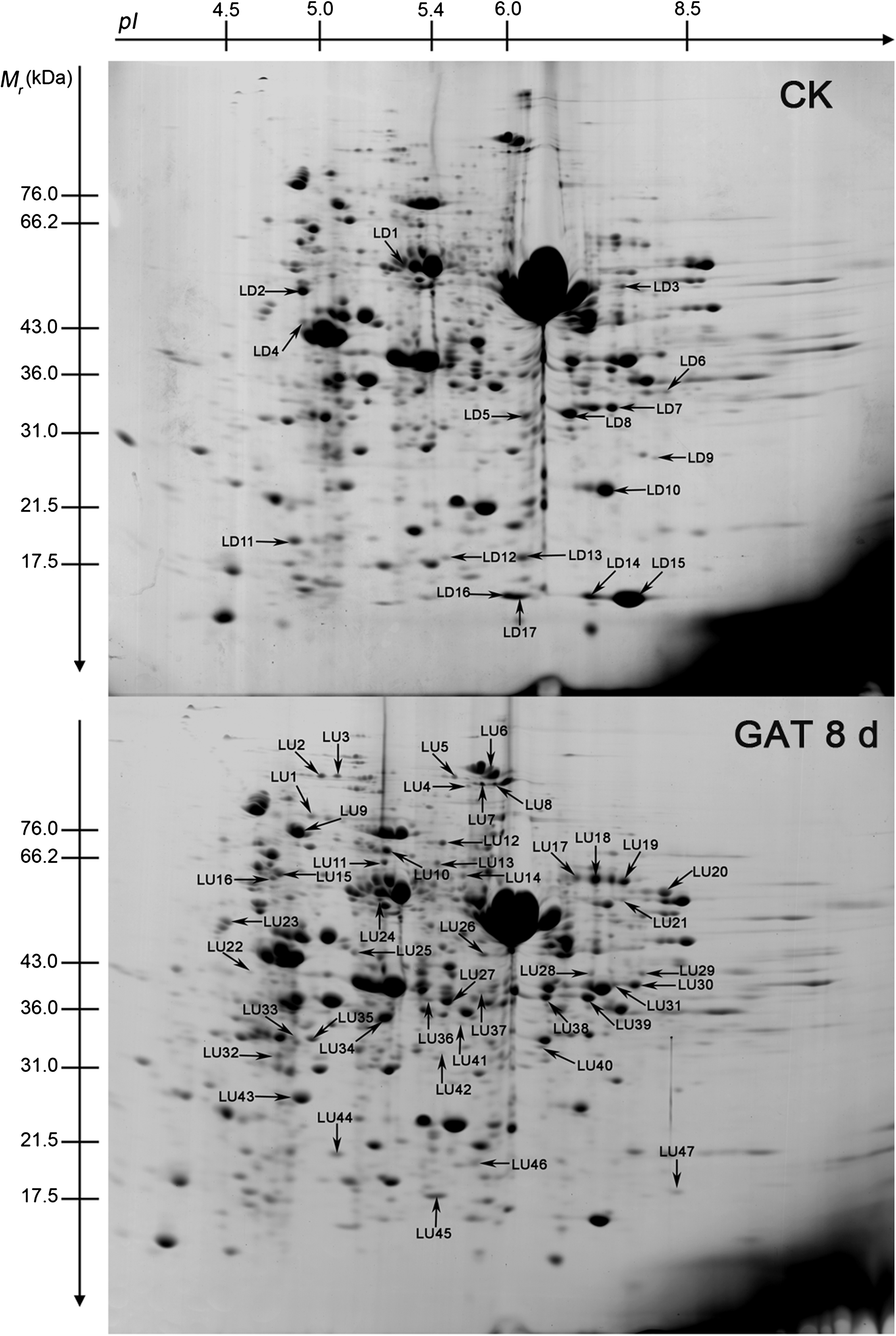

Supplement: Supplementary file 4 — Authors’ original file for figure 3 [file 12284_2013_54_MOESM4_ESM.tif]

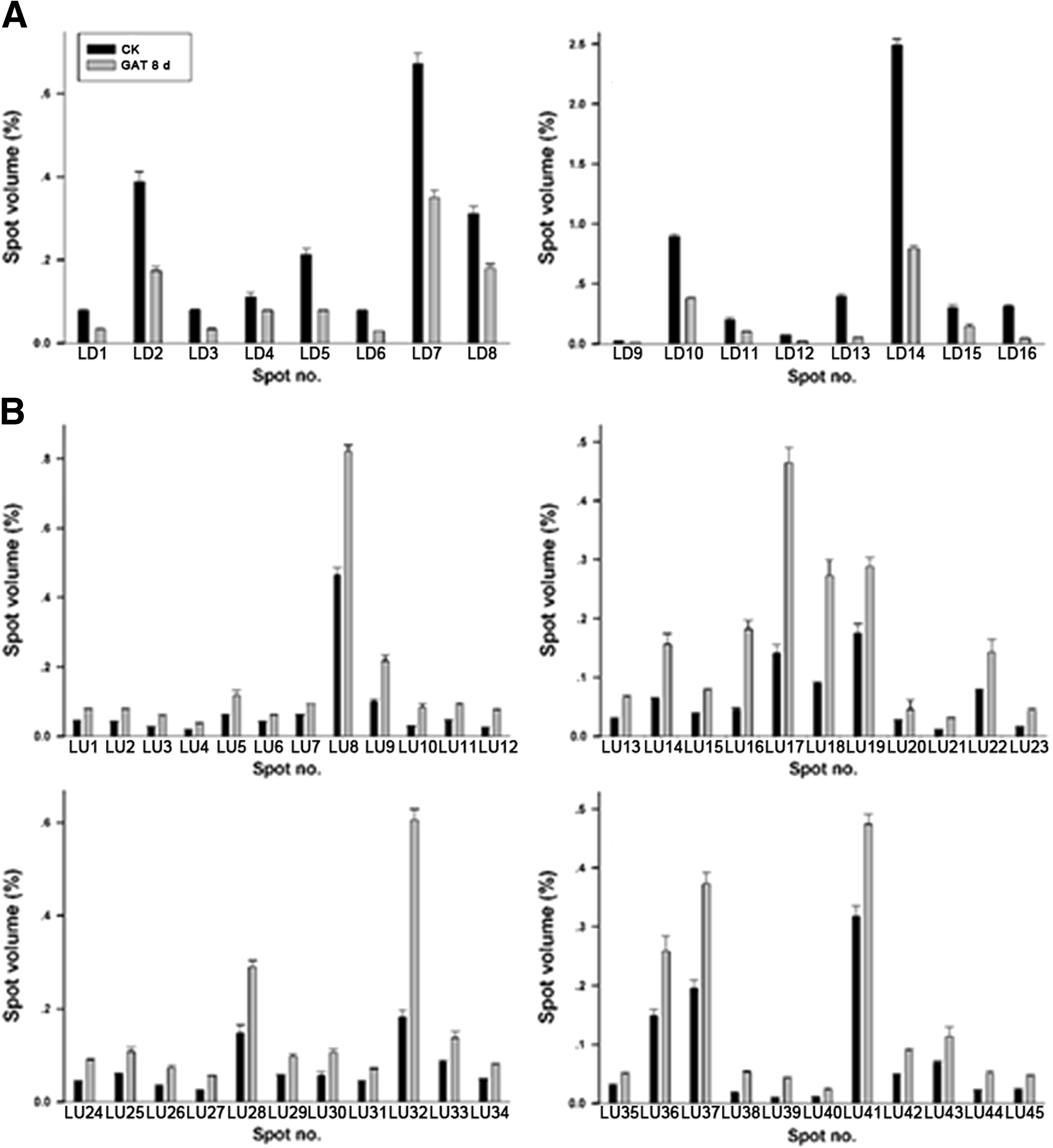

Supplement: Supplementary file 5 — Authors’ original file for figure 4 [file 12284_2013_54_MOESM5_ESM.tif]

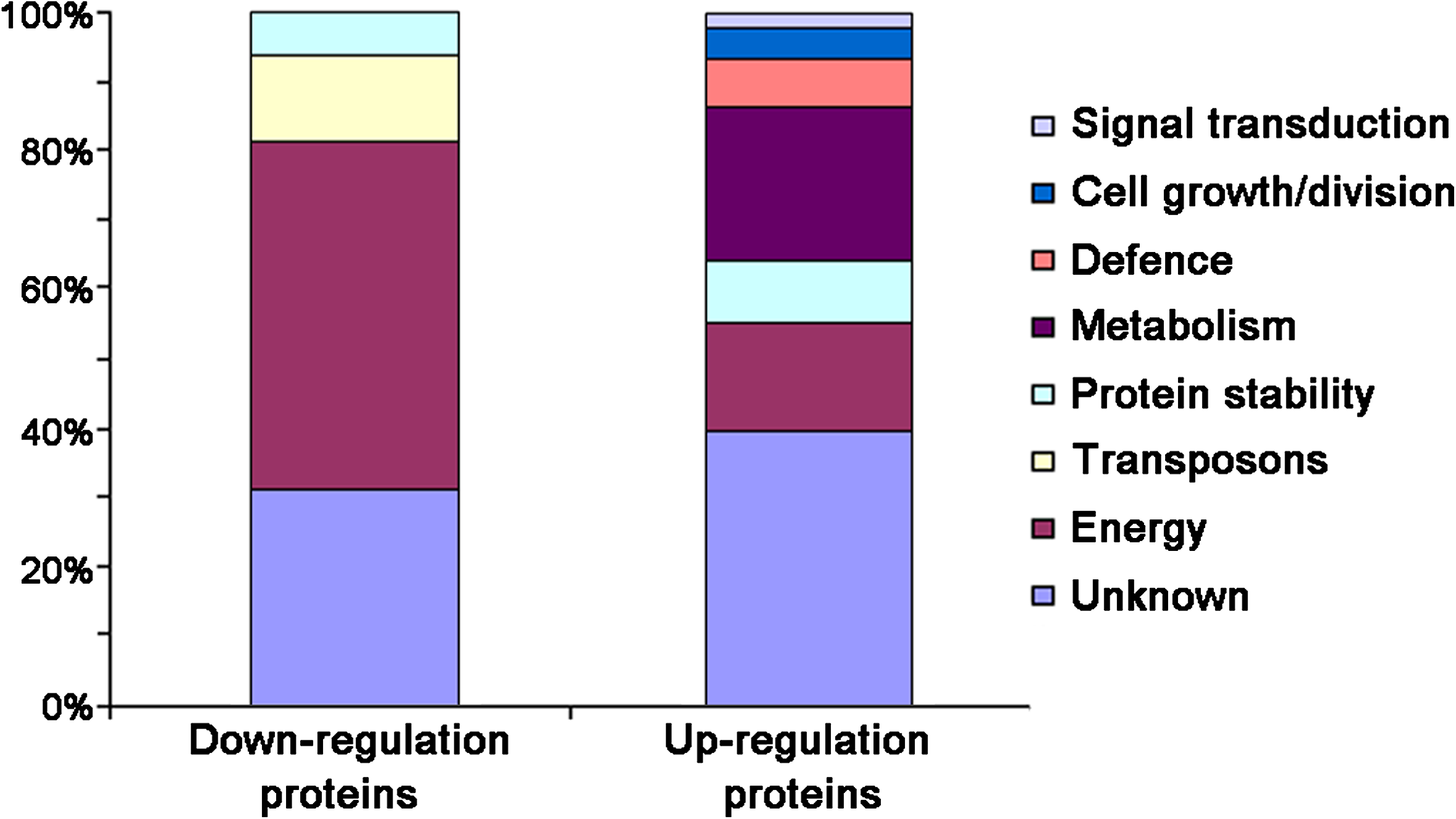

Supplement: Supplementary file 6 — Authors’ original file for figure 5 [file 12284_2013_54_MOESM6_ESM.tif]

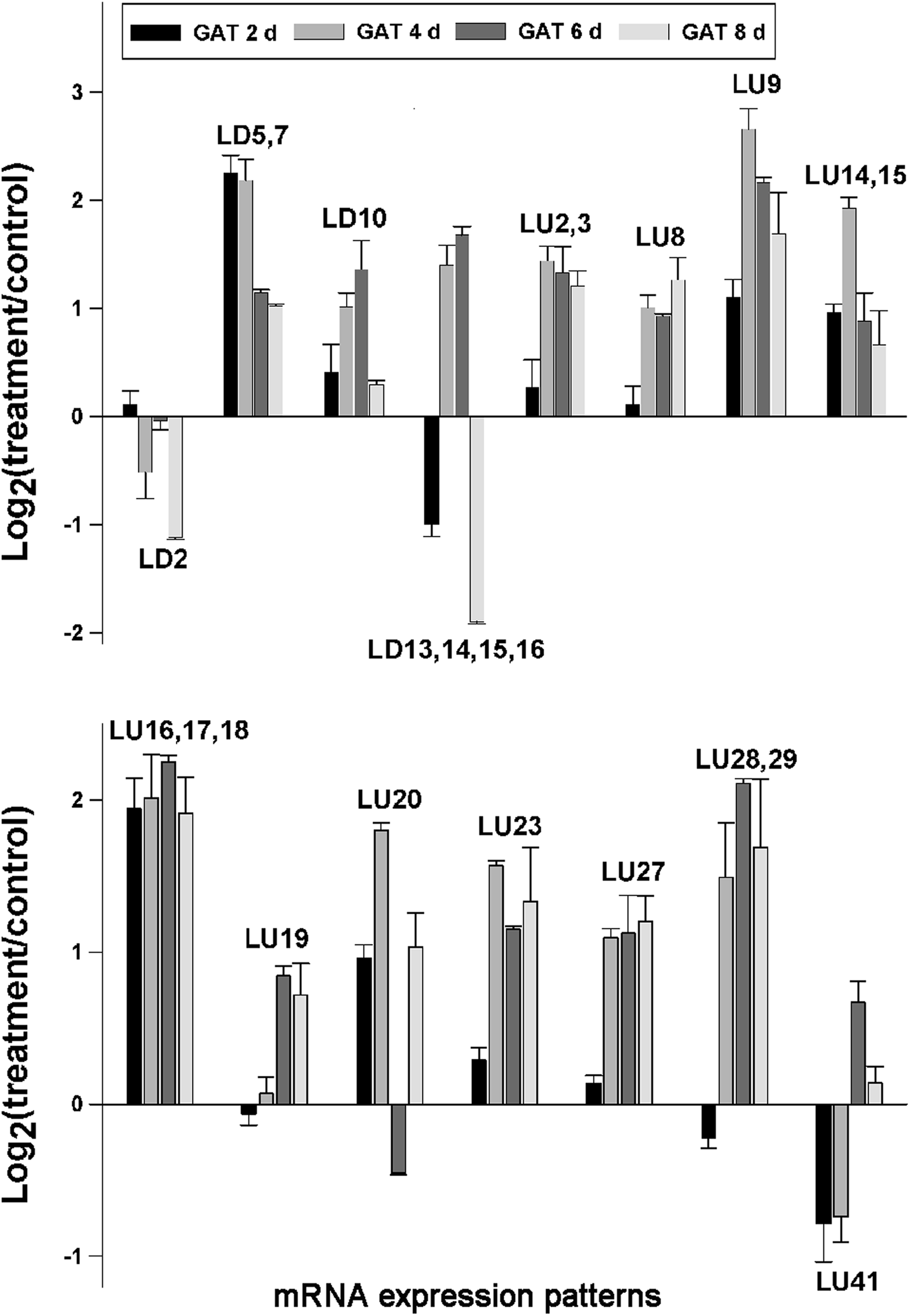

Supplement: Supplementary file 7 — Authors’ original file for figure 6 [file 12284_2013_54_MOESM7_ESM.tif]

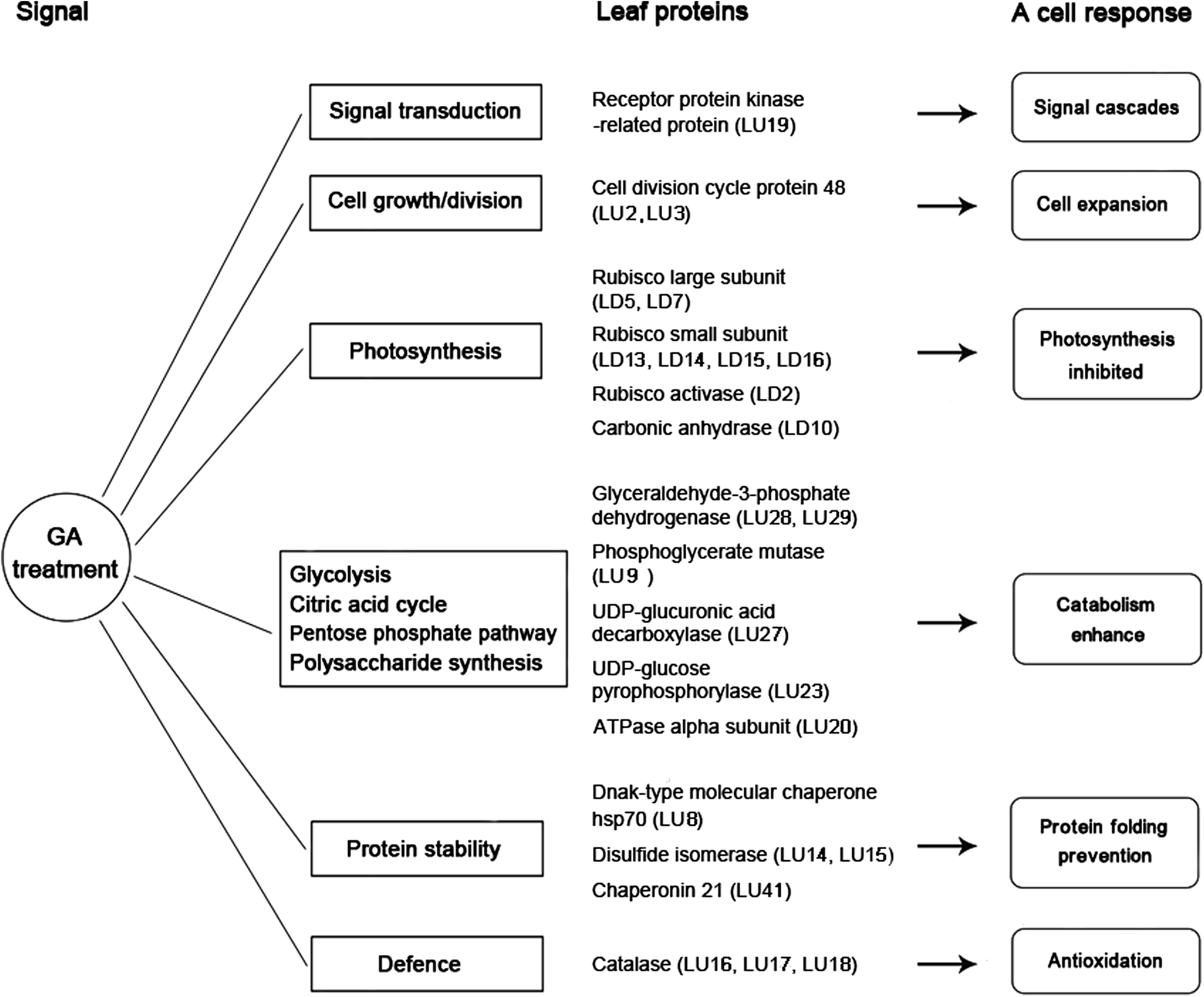

Supplement: Supplementary file 8 — Authors’ original file for figure 7 [file 12284_2013_54_MOESM8_ESM.tif]
